# Clinico-Epidemiological Characteristics of Symptomatic and Asymptomatic Enterotoxigenic and Enteropathogenic *Escherichia Coli* Diarrhea and Impact on Child Growth

**DOI:** 10.4269/ajtmh.24-0347

**Published:** 2025-05-13

**Authors:** Al-Afroza Sultana, Rina Das, Md Nasif Hossain, Rukaeya Amin Sobi, Farina Naz, Soroar Hossain Khan, Sabiha Nasrin, Sharika Nuzhat, Mohammod Jobayer Chisti, Pradip K. Bardhan, Protim Sarker, Tahmeed Ahmed, Subhra Chakraborty, ASG Faruque

**Affiliations:** ^1^ Nutrition Research Division, International Centre for Diarrheal Disease Research, Bangladesh, Dhaka, Bangladesh;; ^2^ Gangarosa Department of Environmental Health, Rollins School of Public Health, Emory University, Atlanta, Georgia;; ^3^ Environmental Institute, University of Virginia, Charlottesville, Virginia;; ^4^ Division of Infectious Diseases and International Health, University of Virginia School of Medicine, Charlottesville, Virginia;; ^5^ Department of Biostatistics and Epidemiology, School of Public Health and Health Sciences, University of Massachusetts, Amherst, Massachusetts;; ^6^ James P. Grant School of Public Health, BRAC University, Dhaka, Bangladesh;; ^7^ Department of Global Health, University of Washington, Seattle, Washington;; ^8^ Department of International Health, Johns Hopkins Bloomberg School of Public Health, Johns Hopkins University, Baltimore, Maryland

## Abstract

Enterotoxigenic *Escherichia* (*E.*) *coli* (ETEC) and enteropathogenic *E. coli* (EPEC) are major bacterial causes of childhood diarrhea. We explored the clinico-epidemiological characteristics of children aged <5 years associated with moderate-to-severe diarrhea (MSD), asymptomatic ETEC or EPEC infections, and subsequent impact on growth reflected by z-score. Data from 9,439 MSD and 13,128 asymptomatic children were extracted from Global Enteric Multicenter Study, conducted between 2007 and 2011. Epidemiological risk factors and clinical characteristics of ETEC and EPEC infection were explored using multivariable logistic regression, and a paired *t*-test was used to investigate the impact of infection on nutritional status. Children aged 12–23 months were more affected by ETEC-positive MSD compared with 0–11 months, whereas children aged 0–11 months were more vulnerable to EPEC-positive MSD. ETEC- and EPEC-positive MSD children showed more characteristics of clinical dehydration like sunken eyes and loss of skin turgor preservation, and needed more intravenous rehydration than ETEC- and EPEC-negative MSD children. Among the other identified co-pathogen, presence of *Campylobacter* in the analyzed stool sample had higher likelihood to be associated with symptomatic MSD (adjusted odds ratio [aOR] 1.42, 95% CI 1.17–1.71) and asymptomatic children with ETEC infection (aOR 1.42, 95% CI 1.16–1.73) and asymptomatic EPEC infection (aOR 1.22, 95% CI 1.04–1.43). Significant growth faltering was noted in MSD children with ETEC (mean difference 0.22, 95% CI 0.10–0.34) and EPEC (mean difference 0.15, 95% CI 0.03–0.27) from baseline to ∼60 days (50–90 days). Our findings highlight the need to implement preventative strategies to reduce the risk of diarrheal illnesses.

## INTRODUCTION

Morbidity, mortality, and poor nutritional outcomes of young children because of diarrheal illnesses are leading health concerns particularly in low- and middle-income countries (LMICs).^1^ According to the World Health Organization (WHO), diarrhea is a leading cause of malnutrition and the third leading cause of death in children aged <5 years, accounting for nearly half a million deaths in this age group each year.^2^ Of these diarrheal deaths, ∼90% occur in South Asia and sub-Saharan Africa.^3^ Enterotoxigenic *Escherichia* (*E.*) *coli* (ETEC) and enteropathogenic *E. coli* (EPEC) are the foremost pathotypes of *E. coli* associated with childhood diarrheal illnesses.^4^ According to the Global Enteric Multicenter Study (GEMS), *Shigella* and heat stable (ST)-toxin producing ETEC, either alone or along with heat labile (LT)-toxin producing ETEC, are the leading enteric pathogens associated with moderate-to-severe diarrhea (MSD) in children aged <5 years.^5^ Pathogens associated with increased risk of infant mortality were ST- ETEC and typical EPEC and the risk of mortality was 8.5 times higher in MSD children than the asymptomatic children within the first 2 years of life.^6^^,^^7^ Apart from diarrheal morbidity and mortality, ETEC and EPEC also have been reported to be linked with other symptoms or conditions caused by undernutrition, especially stunting, among children aged <5 years.^8^^,^^9^ Both ST-ETEC and LT-ETEC develop increasing virulence because of colonization and adherence to the intestinal mucosal membrane which results in profuse loss of water and electrolytes leading to exacerbation of dehydrating diarrheal illnesses.^10^^,^^11^ On the other hand, EPEC produces non-Shiga toxins and causes diarrhea by attaching to and effacing the gut mucosa. An EPEC infection results in abundant watery diarrhea and occasionally bloody diarrhea with mucus, whereas vomiting and low-grade fever are common in infants. Additionally, EPEC causes prolonged diarrhea (≥7 days) or persistent diarrhea (≥14 days), and children with prolonged or persistent diarrhea are more susceptible to growth faltering when compared with children with acute diarrheal episodes.^4^^,^^11^ Given the global burden of ETEC and EPEC strains and the absence of any licensed vaccines,^12^ it is essential to find the clinical and epidemiological risk factors of ETEC and EPEC infections to mitigate diarrheal disease burden and minimize long-term nutritional consequences. The clinical presentation of *E. coli* diarrhea and its effect on malnutrition are quite evident, and the GEMS database gives the unique opportunity to illustrate epidemiology and clinical representations of ETEC and EPEC diarrhea in three age groups (0–11 months, 12–23 months, and 24–59 months) to justify the existing knowledge gap.^11^ In the present study, we investigate the association of socioeconomic characteristics (breast-feeding; clinical features; existing water, sanitation, and hygeine [WASH] conditions and practice) with childhood ETEC and EPEC infection. We also analyze the effect of MSD episodes and asymptomatic infections because of ETEC and EPEC in z-scores of the children studied under 5 years of age.

### Ethical consideration.

Before implementing the GEMS, the local site-specific ethics committees and the University of Maryland School of Medicine ethics committee approved the clinical procedure, consent forms, case-report forms, field procedures, and other study-related supporting materials. The signed informed consents for the children’s participation in the study were obtained from their parents/guardians.

## MATERIALS AND METHODS

### Study sites.

**Figure 1.**
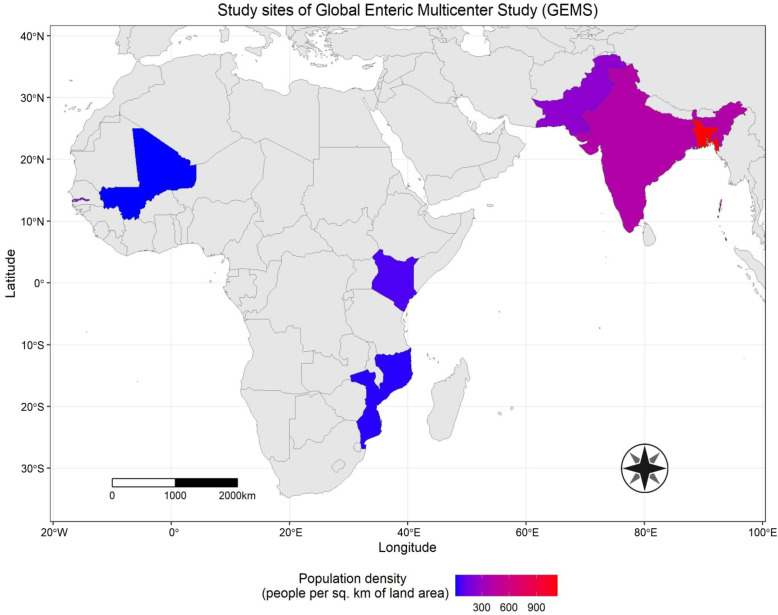
Study sites (data source of population density: World Development Indicators, World Bank).

The GEMS was conducted from December 2007 to March 2011 in seven study sites from South Asia (Bangladesh, India, and Pakistan) and sub-Saharan Africa (the Gambia, Mali, Mozambique, and Kenya)^6^^,^^13^ (Figure 1).

### Study design and participants.

**Figure 2.**
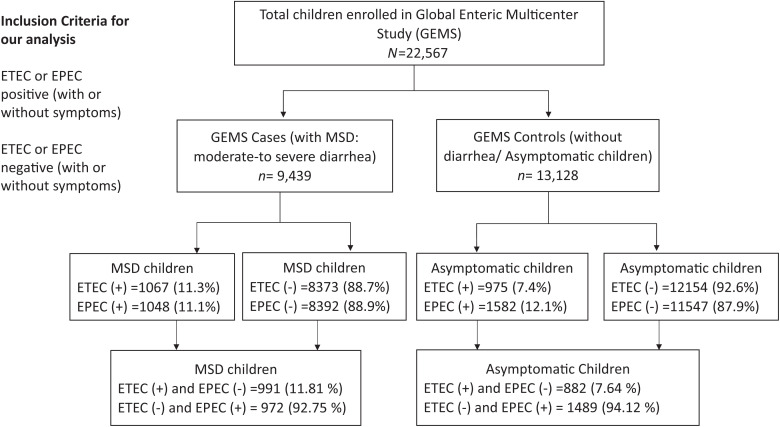
Study flow diagram.

The GEMS was a 3-year, prospective, age-stratified, matched case-control study. Cases were selected from the Demographic Surveillance System area, and only admitted in sentinel health facilities within 7 days of acute illness with MSD. Age- and community-matched asymptomatic children without diarrhea were randomly selected from the same or nearby community as controls within 14 days of the selection of cases (case:control = 1:3). Caregivers of all enrolled MSD and asymptomatic study children were interviewed for the collection of basic information, as well as appraisal of their health and nutritional status based on weight, length/height, and mid-upper arm circumference (MUAC) at the time of enrollment, as well as during the ∼60-day single follow-up visit. For our analysis, data of 9,439 children <5 years of age under-five children with MSD (cases) and 13,128 asymptomatic children (controls) were extracted from GEMS. We included the children whose stool samples were tested and results were indicative of presence or absence of ETEC and EPEC (Figure 2). Detailed study design and methodology of GEMS are described earlier.^6^^,^^7^^,^^13^

### Stool sample collection and fecal microbiology.

As a part of the GEMS, stool samples from cases and controls were collected within 6 hours of evacuation and transported via modified Cary-Blair transport medium and buffered-glycerol saline to a peripheral laboratory. The samples were delievered as early as possible to the central laboratory for plating within 24 hours. In the laboratory, stool was cultured in MacConkey Agar Plates to isolate *E. coli* following conventional methods, and the colonies were pooled and explored using multiplex polymerase chain reaction (PCR) to detect desired pathotypes of *E. coli* including ETEC and EPEC.^14^

### Outcome variable.

Primary outcome variables were ETEC and EPEC (both MSD and asymptomatic) infection among children <5 years of age. We assessed the clinical features (sunken eyes, loss of skin turgor, intravenous [IV] rehydration, dysentery, hospitalization, fever, and vomiting) of EPEC- and ETEC-associated diarrhea, and also measured the effect of ETEC and EPEC on growth by assessing changes of height/length-for-age z-score (HAZ/LAZ), weight-for-age z-score (WAZ), and weight-for-height z-score (WHZ) from baseline to ∼60 days of follow-up.^13^

### Independent variable of interest.

Explanatory variables were epidemiologic features including sociodemographic factors (age, gender, wealth index, primary caretaker, mother’s education, and breastfeeding practice), WASH practices (main source of drinking water, water treatment method, sanitation facility, and handwash practices of caretaker), and co-pathogens (*Giardia, Campylobacter, Aeromonas*, and *Shigella*) of ETEC and EPEC infection among children <5 years.

### Anthropometry.

In the GEMS, simple measures for assessments of nutritional status included length/height, weight, and MUAC. Height and weight of the child were measured at enrollment, after rehydration (in case of MSD), at discharge, and during the ∼60-day follow-up visit.^15^ Length/height was measured three times for each child and later on, the average was computed.^6^^,^^7^^,^^13^ The LAZ/HAZ, WAZ, and WHZ/WLZ were calculated utilizing a WHO statistical analysis system macro using the WHO child growth standards as a reference population.^16^

### MSD.

The status of MSD was defined as new and acute onset of diarrhea (passage of three or more abnormally loose or watery stools in a 24-hour period) within the previous 7 days with at least one of the following preset criteria: 1) sunken eyes (more than normal), 2) decreased skin elasticity (abdominal skin pinch takes a while [>2 seconds] to return to normal), 3) the child either received IV rehydration or prescribed because of acute illness, 4) sought care for dysentery (visible blood in loose stool observed by study staff or reported by mother), and 5) child required hospitalization.^6^^,^^7^^,^^13^

### Asymptomatic children.

Children matched for age, gender, and community, without any diarrhea in the previous 7 days, residing in the same or nearby community were enrolled as controls within 14 days of enrollment of the case.^6^^,^^7^^,^^13^

### Vomiting, fever, and dysentery.

Vomiting was captured as three or more times in a day.

Fever was defined at least 38°C (axillary temperature using digital thermometer) on admission or parental perception. Dysentery was defined as presence of visible blood in loose stool according to the caretaker or the clinician, and determined retrospectively.^6^^,^^7^^,^^13^

### Breastfeeding.

Breastfeeding was captured as whether the child is either exclusively or partially breastfed.^6^^,^^7^^,^^13^

### Sociodemographic information.

Sociodemographic information included the mother as a primary caretaker; and education of mother, categorized as illiterate or literate. The wealth index signifies a household’s collective living standard and were categorized, respectively, from top to bottom in 20% increments as rich, upper-middle, middle, lower middle, and the bottom as poor (but the definition of poor did not follow any usual definition of poverty).^6^^,^^7^^,^^13^ The survey also included WASH data including main source of drinking water (either tube or non-tube well); treated water (used or not); sanitation facility (commonly used by family members for disposal of human fecal excreta and described as sanitary or non-sanitary); use of handwashing materials by primary caretaker (water with or without soap); and detailed handwashing practice (e.g., after defecation, after cleaning a child who defecated, before nursing, before preparing food for household children, and before cooking).^6^^,^^7^^,^^13^

## STATISTICAL ANALYSES

Descriptive analysis was performed for baseline characteristics using frequency and percentage for categorical variables, and mean and SD for continuous variables. The paired *t*-test was used to determine the statistical significance of an observed difference between baseline and end line (∼60 day follow-up after enrollment) z-scores among the study children. Odds ratios (ORs) and 95% CIs were calculated to measure the strength of the association between ETEC or EPEC infection and the independent variables of interest. The covariates included age, gender, breastfeeding status, primary caretaker’s education, WASH, and co-pathogens *(Giardia, Campylobacter, Aeromonas,* and *Shigella).* The z-scores for HAZ/LAZ, WAZ, and WHZ were adjusted for continuous variables. Multivariable logistic regression analyses were performed after adjusting for all the covariates. A *P*-value less than 0.05 was considered statistically significant. In regression models, any covariate associated with a level of significance *P* <0.25 was retained but statistical significance in the final model was considered as *P* <0.05.^17^ Other relevant variables such as age and gender were included in the model because of their biological, as well as public, health importance according to the literature review. Data management and analyses were undertaken by STATA (Version 16), Epi Info (Version 7.0), and R (version 4.2.0).

## RESULTS

The findings obtained from our analyses came from 9,439 MSD and 13,128 asymptomatic children. Of the MSD children, 1,067 (11.3%) were ETEC-positive and 1,048 (11.1%) were EPEC-positive (Table 1). The number of asymptomatic children with ETEC infection was 975 (7.4%) and with EPEC infection was 1,582 (12.1%) (Table 2).

**Table 1:** Baseline characteristics of the MSD children with or without ETEC and EPEC infection

Characteristics of Children	MSD (*N*) = 9,439(%)					
	ETEC Positive *n* = 1,067 (11.3%)	ETEC Negative *n* = 8,373 (88.7%)	*P*-Value	EPEC Positive *n* = 1,048 (11.1%)	EPEC Negative *n* = 8,391 (88.9%)	*P*-Value
Age						
0–11 months	427 (40.0)	3,603 (43.0)	<0.001	512 (48.9)	3,518 (41.9)	<0.001
12–23 months	424 (39.7)	2,781 (33.2)		328 (31.3)	2,877 (34.3)	
24–59 months	216 (20.2)	1,989 (23.8)		208 (19.9)	1,997 (23.8)	
Gender (girl)	462 (43.3)	3,633 (43.4)	0.955	455 (43.4)	3,640 (43.4)	0.980
Breastfed	746 (69.9)	5,995 (71.6)	0.252	769 (73.4)	5,972 (71.2)	0.135
Baseline anthropometry						
HAZ*	−1.4 ± 1.4	−1.3 ± 1.4	0.021	−1.4 ± 1.4	−1.3 ± 1.4	0.299
WAZ*	−1.7 ± 1.5	−1.5 ± 1.4	<0.001	−1.6 ± 1.5	−1.5 ± 1.4	0.004
WHZ*	−1.3 ± 1.6	−1.0 ± 1.5	<0.001	−1.2 ± 1.5	−1.0 ± 1.5	0.014
Sociodemographic characteristics						
Primary caretaker (mother)	1,021 (95.7)	8,033 (95.9)	0.697	998 (95.2)	8,056 (96.0)	0.237
Mother’s education						
Literate	553 (52.1)	4,833 (57.9)	<0.001	565 (54.1)	4,821 (57.7)	0.028
Illiterate	508 (47.9)	3,508 (42.1)	<0.001	479 (45.9)	3,537 (42.3)	0.028
Wealth index						
Richest	194 (18.1)	1,627 (19.5)	0.299	199 (19.0)	1,622 (19.3)	0.910
Upper middle	189 (17.7)	1,591 (19.0)		196 (18.7)	1,584 (18.9)	
Middle	218 (20.4)	1,775 (21.2)		212 (20.3)	1,781 (21.2)	
Lower middle	226 (21.1)	1,587(18.9)		208 (19.9)	1,605 (19.1)	
Poor	240 (22.5)	1,787 (21.4)		232 (22.1)	1,795 (21.4)	
WASH						
Main source of drinking water						
Tube well	122 (11.4)	1,553 (18.6)	<0.001	161 (15.4)	1,514 (18.0)	0.032
Non-tube well	945 (88.6)	6,820 (81.5)	<0.001	887 (84.6)	6,878 (81.9)	0.032
Water treatment method						
Use treated water	346 (32.4)	2,353 (28.1)	0.003	320 (30.5)	2,379 (28.4)	0.140
Untreated water	721 (67.6)	6,020 (71.9)	0.003	728 (69.5)	6,013 (71.6)	0.140
Sanitation facility						
Improved	1,013 (94.9)	7,966 (95.1)	0.775	994 (94.9)	7,985 (95.2)	0.668
Unimproved	54 (5.1)	407 (4.9)	0.775	54 (5.2)	407 (4.9)	0.668
Handwashing						
With soap	806 (75.5)	6,325 (75.6)	0.994	809 (77.2)	6,322 (75.3)	0.188
Without soap	261 (24.4)	2,047 (24.5)	0.994	239 (22.8)	2,069 (24.7)	0.188
WASH practice (caretaker)						
Handwashing after defecation	762 (71.4)	6,256 (74.7)	0.020	776 (74.1)	6,242 (74.4)	0.815
After cleaning a child who defecated	513 (48.1)	3,736 (44.6)	0.032	449 (42.8)	3,800 (45.3)	0.135
Before nursing a child or preparing baby’s food	414 (38.8)	3,269 (39.0)	0.879	414 (39.5)	3,269 (38.9)	0.731
Before cooking	616 (57.7)	5,143(61.4)	0.020	624 (59.5)	5,135 (61.2)	0.303
Common co-pathogens isolated in stool						
*Giardia*	205 (19.2)	1,581 (18.9)	0.798	207 (19.8)	1,579 (18.8)	0.468
*Campylobacter*	163 (15.3)	1,008 (12.0)	0.003	139 (13.3)	1,032 (12.3)	0.372
*Aeromonas*	54 (5.0)	601 (7.2)	0.010	58 (5.5)	597 (7.1)	0.058
*Shigella*	73 (6.8)	1,037(12.4)	<0.001	113 (10.8)	997 (11.9)	0.298
Clinical features						
Sunken eyes	956 (89.6)	6,548 (78.2)	<0.001	853 (81.4)	6,651 (79.3)	0.106
Loss of skin turgor	254 (23.8)	1,712 (20.5)	0.011	281 (26.8)	1,685 (20.1)	<0.001
Vomiting ≥3 times	453 (42.5)	3,193 (38.1)	0.022	432 (41.2)	3,214 (38.3)	0.166
IV rehydration	182 (17.1)	1,238 (14.8)	0.049	186 (17.8)	1,234 (14.7)	0.009
Dysentery	121 (11.3)	1,887 (22.5)	<0.001	204 (19.5)	1,804 (21.5)	0.006
Fever	611 (57.3)	5,164 (61.7)	0.019	654 (62.4)	5,121 (61.3)	0.682
Required hospitalization	190 (17.8)	1,483 (17.7)	0.939	195 (18.6)	1,478 (17.6)	0.427

EPEC = enteropathogenic *Escherichia coli*; ETEC = enterotoxigenic *Escherichia coli*; HAZ = height-for-age z-score; MSD = moderate-to-severe diarrhea; WASH = water, sanitation, and hygiene; WAZ = weight-for-age z-score; WHZ = weight-for-height z-score.

* Data are presented as mean ± SD for heat-for-age z-score, weight-for-age z-score, and weight-for-height z-score.

**Table 2:** Baseline characteristics of the asymptomatic children with or without ETEC and EPEC infection

Characteristics of Children	Asymptomatic (*N*) = 13,128 (%)					
	ETEC Positive *n* = 975 (7.4%)	ETEC Negative *n* = 12,154 (92.6%)	*P*-Value	EPEC Positive *n* = 1,582 (12.1%)	EPEC Negative *n* = 11,547 (87.9%)	*P*-Value
Age						
0–11 months	360 (36.9)	4,518 (37.2)	<0.001	645 (40.8)	4,233 (36.7)	<0.001
12–23 months	393 (40.3)	3,988 (32.8)		543 (34.3)	3,838 (33.3)	
24–59 months	222 (22.8)	3,648 (30.0)		394 (24.9)	3,476 (30.1)	
Gender (girl)	416 (42.7)	5,235 (43.1)	0.806	684 (43.2)	4,967 (43.0)	0.868
Breastfed	689 (70.7)	8,350 (68.7)	0.202	1,151 (72.8)	7,888 (68.3)	<0.001
Baseline anthropometry						
HAZ*	−1.4 ± 1.3	−1.3 ± 1.3	0.535	−1.3 ± 1.3	−1.4 ± 1.3	0.068
WAZ*	−1.0 ± 1.3	−1.1 ± 1.3	0.363	−1.0 ± 1.3	−1.1 ± 1.3	0.080
WHZ*	−0.4 ± 1.5	−0.5 ± 1.4	0.326	−0.4 ± 1.3	−0.5 ± 1.4	0.357
Sociodemographic characteristics						
Primary caretaker (mother)	943 (96.7)	11,768 (96.8)	0.856	1,526 (96.5)	11,185 (96.9)	0.390
Mother’s education						
Literate	541 (55.6)	7,394 (60.9)	0.001	973 (61.6)	6,962 (60.4)	0.357
Illiterate	432 (44.4)	4,736 (39.0)	0.001	606 (38.4)	4,562 (39.6)	0.357
Wealth index						
Richest	182 (18.7)	2,490 (20.5)	0.010	296 (18.7)	2,376 (20.6)	0.203
Upper middle	186 (19.1)	2,336 (19.2)		318 (20.1)	2,204 (19.1)	
Middle	254 (26.1)	2,580 (21.2)		332 (20.9)	2,502 (21.7)	
Lower middle	179 (18.4)	2,411 (19.8)		338 (21.4)	2,252 (19.5)	
Poor	173 (17.8)	2,337 (19.2)		298 (18.8)	2,212 (19.2)	
WASH						
Main source of drinking water						
Tube well	161 (16.5)	2,833 (23.3)	<0.001	354 (22.4)	2,640 (22.9)	0.665
Non-tube well	814 (83.5)	9,321 (76.7)	<0.001	1,228 (77.6)	8,907 (77.1)	
Water treatment method						
Use treated water	227 (23.3)	2,645 (21.8)	0.269	372 (23.5)	2,500 (21.7)	0.093
Untreated water	748 (76.7)	9,509 (78.2)	0.269	1,210 (76.5)	9,047 (78.4)	0.093
Sanitation facility						
Improved	910 (93.3)	11,379 (93.6)	0.722	1,503 (95.0)	10,786 (93.4)	0.015
Unimproved	65 (6.7)	775 (6.4)	0.722	79 (4.9)	761 (6.6)	0.015
Handwashing						
With soap	727 (74.6)	9,035 (74.3)	0.838	1,211 (76.6)	8,551 (74.1)	0.034
Without soap	247 (25.4)	3,118 (25.7)	0.838	371 (23.4)	2,994 (25.9)	0.034
WASH practice (caretaker)						
Handwashing after defecation	708 (72.6)	9,013 (74.2)	0.291	1,183 (74.8)	8,538 (73.9)	0.476
After cleaning a child who defecated	472 (48.4)	5,717 (47.0)	0.409	765 (48.4)	5,424 (46.9)	0.301
Before nursing a child or preparing baby’s food	387 (39.7)	4,746 (39.0)	0.692	666 (42.1)	4,467 (38.7)	0.009
Before cooking	638 (65.4)	8,499 (69.9)	0.003	1,094 (69.2)	8,043 (69.7)	0.684
Co-pathogens isolated in stool						
*Giardia*	273 (28.0)	3,197 (26.3)	0.249	426 (26.9)	3,044 (26.4)	0.633
*Campylobacter*	137 (14.1)	1,424 (11.7)	0.030	211 (13.3)	1,350 (11.7)	0.058
*Aeromonas*	37 (3.8)	581 (4.8)	0.162	80 (5.1)	538 (4.7)	0.484
*Shigella*	11 (1.1)	220 (1.8)	0.119	27 (1.7)	204 (1.8)	0.865

EPEC = enteropathogenic *Escherichia coli*; ETEC = enterotoxigenic *Escherichia coli*; HAZ = height-for-age z-score; WASH = water, sanitation, and hygiene; WAZ = weight-for-age z-score; WHZ = weight-for-height z-score.

* Data are presented as mean ± SD for heat-for-age z-score, weight-for-age z-score, and weight-for-height z-score.

### Distribution of ETEC and EPEC infection among seven GEMS sites.

**Figure 3.**
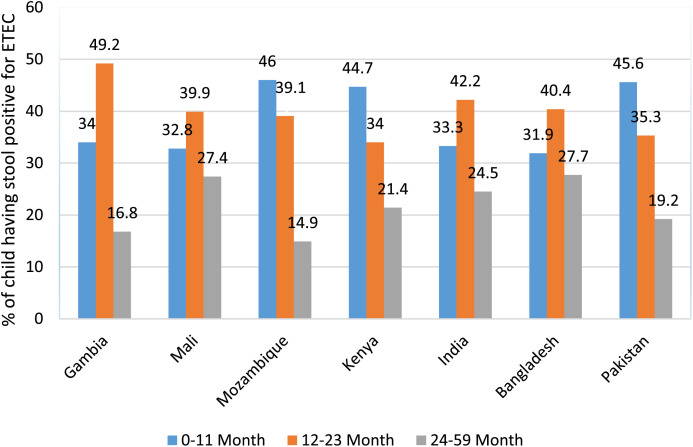
Site- and age-specific distribution of enterotoxigenic *Escherichia coli* (ETEC) diarrhea in seven sites of Global Enteric Multicenter Study (GEMS).

**Figure 4.**
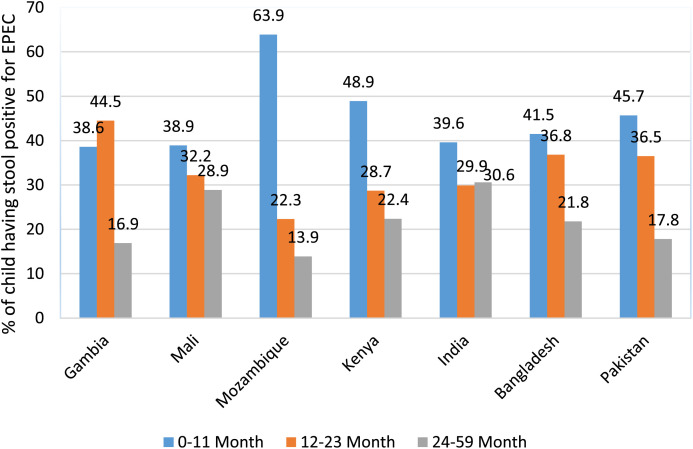
Site- and age-specific distribution of enteropathogenic *Escherichia coli* (EPEC) diarrhea in seven sites of Global Enteric Multicenter Study (GEMS).

Among all GEMS sites, ETEC was isolated mostly in Gambia (49%), India (42%), and Bangladesh (40%) in the 12–23 months age group. Children with ETEC in the 0–11 months age group were affected more in Mozambique (46%), Pakistan (46%), and Kenya (45%) (Figure 3). More EPEC-infected cases among the 0–11 months age group were in Mozambique (64%), Kenya (49%), Pakistan (46%), and Bangladesh (41%), and those in the 12–23 months group were in Gambia (45%), Bangladesh (37%), and Pakistan (36%). Other sites had less EPEC episodes. ETEC-infected cases among the 24–59 months age group were more often reported in Bangladesh (28%), Mali (27%) (Figure 4), and India (24%), and EPEC episodes were more common in 24–59 months group in India (31%), Mali (29%), Kenya (22%), and Bangladesh (22%). Other sites had less ETEC- and EPEC-infected cases (Figures 3, 4).

### Characteristics of ETEC- and EPEC-positive MSD children.

ETEC- and EPEC-infected children (with MSD and without symptoms) had nearly similar distribution in different age groups (0–11 months, 12–23 months, and 24–59 months), genders, and breastfeeding statuses (Tables 1 and 2). ETEC-positive MSD was found predominantly in children aged 0–11 months and 12–23 months (about 40% in each age group) and EPEC-positive MSD was found in the 0–11 months age group (49%). Girls (43%) were less susceptible to ETEC and EPEC MSD than boys. At the time of enrollment children with ETEC-positive MSD had more severe key clinical features than ETEC-negative MSD children, such as sunken eyes, loss of skin turgor, vomiting, and need for IV rehydration therapy. EPEC-positive MSD children also experienced loss of skin turgor and IV rehydration more often than EPEC-negative MSD. Blood in stool and fever were more frequently observed in ETEC-negative MSD children than in ETEC-positive MSD children. Mothers were more literate in the case of ETEC- and EPEC-negative children than positive MSD children, and often their drinking water source was a tube well. However, the use of treated water was higher in ETEC-positive than negative MSD. Handwashing after defecation and before cooking was lower among caregivers of ETEC-positive than negative MSD, but the opposite was seen for handwashing after cleaning a child who defecated. *Campylobacter* was a common co-pathogen identified in ETEC-positive MSD children (*P* <0.001), and *Aeromonas* and *Shigella* were more often observed as co-pathogens in ETEC-negative children, but this was not statistically significant (Table 1).

### Characteristics of ETEC- and EPEC-positive asymptomatic children.

Approximately 37% and 40% ETEC positivity was found in children aged 0–11 months and 12–23 months, respectively, and EPEC positivity was predominantly found in the 0–11 months age group (41%) in asymptomatic children. Mothers of EPEC-positive asymptomatic children more often practiced breastfeeding than EPEC-negative children but this was not statistically significant. In children aged 24–59 months, non-breastfeeding was associated with higher odds of ETEC-related diarrhea (OR 1.51, *P* = 0.001), suggesting non-breastfeeding may increase susceptibility to ETEC infections in this age group, whereas no significant association was found for EPEC across any age group (Supplemental Table 1). Mothers of asymptomatic ETEC-negative children were more literate than mothers of ETEC-positive asymptomatic children. Tube well as a drinking water source was also more frequently used by the households of ETEC-negative asymptomatic children compared with ETEC-positive asymptomatic children. Handwashing before cooking was practiced more in ETEC-negative households. Use of improved sanitation facilities, soap for handwashing, and handwashing before nursing a child or preparing baby’s food were higher in EPEC-positive than negative households. Detection of *Campylobacter* as a co-pathogen was more often observed in asymptomatic ETEC-positive than ETEC-negative children (Table 2).

### Association of clinico-epidemiological factors with ETEC or EPEC infection among children with MSD and in asymptomatic children.

According to multivariate logistic regression analysis of ETEC cases, children aged 12–23 months had higher odds of having ETEC MSD compared with 0–11 months children (Table 3). For asymptomatic ETEC infection, there was no such age variation. Children from middle-class households showed a higher likelihood of being infected with asymptomatic ETEC compared with the richest household; however, no such association was observed between the wealth index and ETEC MSD (Table 4). The presence of *Campylobacter* in stool samples had a significant positive association with ETEC infection both in asymptomatic and MSD children (Tables 3 and 4). Among clinical features of MSD, sunken eyes had a significant positive association, whereas dysentery and fever had negative associations with ETEC infection (Table 3).

**Table 3:** Association of clinico-epidemiological factors with ETEC and EPEC infection among MSD children

Clinico-Epidemiological Factors	ETEC (+)				EPEC (+)			
	Unadjusted OR (95% CI)	*P*-Value	aOR (95% CI)	*P*-Value	Unadjusted OR (95% CI)	*P*-Value	aOR (95% CI)	*P*-Value
Age group (months)								
0–11	Ref							
12–23	1.28 (1.11–1.48)	0.001	1.28 (1.07–1.53)	0.006	0.78 (0.67–0.90)	0.001	0.79 (0.66–0.95)	0.011
24–59	0.91 (0.77–1.08)	0.321	0.98 (0.77–1.24)	0.852	0.71 (0.60–0.849)	0.000	0.7 (0.55–0.89)	0.003
Gender								
Boy	Ref							
Girl	0.99 (0.87–1.13)	0.955	1.01 (0.89–1.15)	0.873	1.01 (0.87–1.14)	0.980	1.01 (0.88–1.15)	0.944
Breastfed								
Yes	Ref							
No	1.08 (0.94–1.24)	0.252	1.02 (0.85–1.24)	0.798	0.89 (0.77–1.03)	0.135	0.98 (0.81–1.19)	0.817
Baseline anthropometry								
HAZ	0.94 (0.90–0.99)	0.022	0.89 (0.70–1.14)	0.366	0.97 (0.93–1.02)	0.299	1.1 (0.88–1.38)	0.395
WAZ	0.90 (0.86–0.94)	0.000	1.22 (0.80–1.87)	0.354	0.93 (0.90–0.97)	0.004	0.78 (0.54–1.14)	0.197
WHZ	0.89 (0.86–0.93)	0.000	0.78 (0.58–1.05)	0.104	0.94 (0.90, 0.98)	0.014	1.13 (0.88–1.47)	0.341
Sociodemographic characteristics								
Mother’s education								
Literate	Ref							
Illiterate	1.26 (1.13–1.43)	0.000	1.02 (0.86–1.21)	0.845	1.15 (1.01–1.31)	0.028	1.16 (0.98–1.38)	0.088
Wealth index								
Richest	Ref							
Upper middle	0.99 (0.80–1.23)	0.972	0.97 (0.78–1.20)	0.775	1.01 (0.81–1.24)	0.936	1.01 (0.81–1.24)	0.961
Middle	1.03 (0.83–1.26)	0.777	0.93 (0.75–1.15)	0.499	0.97 (0.79–1.19)	0.772	0.95 (0.77–1.17)	0.604
Lower middle	1.19 (0.97–1.46)	0.088	1.09 (0.89–1.35)	0.401	1.05 (0.85–1.29)	0.603	1.03 (0.84–1.28)	0.754
Poor	1.12 (0.92–1.37)	0.245	1.06 (0.86–1.31)	0.576	1.05 (0.86, 1.28)	0.611	1.05 (0.85–1.30)	0.648
WASH								
Source of drinking water								
Tube well	Ref							
Non-tube well	1.76 (1.44–2.14)	0.000	0.82 (0.59–1.14)	0.242	1.21 (1.01–1.44)	0.033	1.37 (0.90–2.07)	0.139
Use of treated water								
Yes	Ref							
No	0.81 (0.71–0.93)	0.003	1.04 (0.90–1.23)	0.563	0.90 (0.78–1.04)	0.140	0.88 (0.74–1.04)	0.15
Sanitation facility								
Yes	Ref							
No	1.043 (0.77–1.39)	0.775	0.85 (0.62–1.17)	0.319	1.06 (0.79–1.42)	0.668	0.91 (0.67–1.25)	0.562
Handwashing								
With soap	Ref							
Without soap	0.98 (0.84–1.14)	0.838	1.02 (0.86–1.19)	0.856	0.90 (0.77–1.05)	0.189	0.96 (0.82–1.14)	0.662
Co-pathogens present in stool								
*Giardia*								
Absent	Ref							
Present	1.02 (0.86–1.20)	0.798	0.95 (0.80–1.13)	0.584	1.06 (0.90–1.24)	0.468	1.17 (0.98–1.39)	0.075
*Campylobacter*								
Absent	Ref							
Present	1.31 (1.10–1.57)	0.003	1.42 (1.17–1.71)	0.000	1.09 (0.90–1.31)	0.372	1.15 (0.95–1.41)	0.159
*Aeromonas*								
Absent	Ref							
Present	0.68 (0.51–0.91)	0.011	0.97 (0.70–1.33)	0.831	0.76 (0.57–1.00)	0.058	0.84 (0.62–1.15)	0.277
*Shigella*								
Absent	Ref							
Present	0.51 (0.40–0.66)	0.000	0.78 (0.59–1.02)	0.073	0.89 (0.72–1.10)	0.298	1.09 (0.86–1.38)	0.484
Clinical features (MSD case)								
Sunken eyes								
No	Ref							
Yes	2.40 (1.95–2.94)	0.000	1.40 (1.04–1.90)	0.028	1.14 (0.97–1.34)	0.106	0.96 (0.73,1.26)	0.765
Loss of skin turgor								
No	Ref							
Yes	1.21 (1.04–1.41)	0.011	0.92 (0.77–1.09)	0.313	1.45 (1.25–1.68)	0.000	1.28 (1.08–1.51)	0.005
Vomiting ≥3 times								
No	Ref							
Yes	1.19 (1.05–1.36)	0.006	1.03 (0.89–1.18)	0.710	1.12 (0.99–1.28)	0.068	1.02 (0.88–1.17)	0.801
Dysentery								
No	Ref							
Yes	0.43 (0.36–0.53)	0.000	0.68 (0.53–0.87)	0.002	0.88 (0.75–1.03)	0.134	1.06 (0.84–1.33)	0.632
Fever								
No	Ref							
Yes	0.83 (0.73–0.94)	0.005	0.83 (0.72–0.95)	0.008	1.05 (0.9–1.20)	0.394	0.98 (0.85–1.13)	0.8
IV rehydration								
No	Ref							
Yes	1.18 (1.01–1.40)	0.049	0.95 (0.76–1.20)	0.682	1.25 (1.05–1.48)	0.010	1.01 (0.80–1.25)	0.977
Hospitalization								
No	Ref							
Yes	1.00 (0.85–1.18)	0.939	0.96 (0.77–1.20)	0.724	1.06 (0.90–1.26)	0.427	1.05 (0.85–1.31)	0.653

Multiple logistic regression was performed for association analysis between dependent and independent variables. The z-score for height-for-age, weight-for-age, and weight-for-height or length were adjusted as continuous variables. We controlled sites as a cluster effect in the multivariate logistics regression model. aOR = adjusted odds ratio; EPEC = enteropathogenic *Escherichia coli*; ETEC = enterotoxigenic *Escherichia coli*; HAZ = height-for-age z-score; IV = intravenous; MSD = moderate-to-severe diarrhea; OR = odds ratio; Ref = reference; WASH = water, sanitation, and hygiene; WAZ = weight-for-age z-score; WHZ = weight-for-height z-score.

**Table 4:** Association of clinico-epidemiological factors with ETEC and EPEC infection among asymptomatic children

Factors	ETEC (+)				EPEC (+)			
	Unadjusted OR (95% CI)	*P*-Value	aOR (95% CI)	*P*-Value	Unadjusted OR (95% CI)	*P*-Value	aOR (95% CI)	*P*-Value
Age group (months)								
0–11	Ref							
12–23	1.23 (1.06–1.43)	0.005	1.09 (0.90–1.31)	0.370	0.92 (0.82–1.04)	0.234	0.90 (0.77–1.04)	0.152
24–59	0.76 (0.64–0.90)	0.002	0.79 (0.62–1.02)	0.066	0.74 (0.65–0.84)	0.000	0.74 (0.61–0.89)	0.001
Sex								
Boy	Ref							
Girl	0.98 (0.86–1.12	0.806	1.01 (0.88–1.15)	0.904	1.00 (0.90–1.12)	0.868	1.02 (0.91–1.13)	0.765
Breastfed								
Yes	Ref							
No	0.91 (0.78–1.05)	0.203	0.95 (0.78–1.16)	0.611	0.807 (0.71–0.90)	0.000	0.97 (0.83–1.13)	0.675
Baseline anthropometry								
HAZ	0.98 (0.93–1.03)	0.535	0.68 (0.52–0.90)	0.007	1.04 (0.99–1.08)	0.068	0.91 (0.74–1.11)	0.352
WAZ	1.02 (0.97–1.07)	0.363	1.91 (1.17–3.12)	0.010	1.03 (0.99–1.07)	0.080	1.18 (0.83–1.68)	0.348
WHZ	1.02 (0.97–1.07)	0.325	0.63 (0.44–0.89)	0.010	1.01 (0.98–1.05)	0.357	0.90 (0.70–1.16)	0.414
Sociodemographic characteristics								
Mother’s education								
Literate	Ref							
Illiterate	1.24 (1.09–1.42)	0.001	1.03 (0.85–1.24)	0.786	0.95 (0.85–1.05)	0.357	0.97 (0.84–1.11)	0.628
Wealth index								
Richest	Ref							
Upper middle	1.08 (0.88–1.34)	0.429	1.09 (0.88–1.35)	0.425	1.15 (0.97–1.37)	0.088	1.16 (0.98–1.37)	0.094
Middle	1.34 (1.10–1.64)	0.003	1.33 (1.08–1.63)	0.006	1.06 (0.90–1.25)	0.457	1.09 (0.92–1.29)	0.305
Lower middle	1.01 (0.82–1.25)	0.886	0.97 (0.78–1.21)	0.81	1.20 (1.02–1.42)	0.028	1.27 (1.07–1.51)	0.006
Poor	1.01 (0.81–1.25)	0.908	0.96 (0.77–1.21)	0.756	1.08 (0.91–1.28)	0.370	1.14 (0.96–1.37)	0.14
WASH								
Source of drinking water								
Tube well	Ref							
Non-tube well	1.53 (1.29–1.82)	0.000	1.06 (0.78–1.42)	0.715	1.02 (0.90–1.16)	0.665	1.12 (0.84–1.49)	0.441
Use of treated water								
Yes	Ref							
No	0.91 (0.78–1.06)	0.270	1.01 (0.84–1.19)	0.952	1.11 (0.98–1.25)	0.093	0.88 (0.76–1.02)	0.096
Sanitation facility								
Yes	Ref							
No	1.04 (0.80–1.36)	0.722	0.93 (0.70–1.25)	0.638	0.74 (0.58–0.94)	0.015	0.77 (0.59–0.99)	0.047
Handwashing								
With soap	Ref							
Without soap	0.90 (0.77–1.05)	0.189	1.08 (0.92–1.27)	0.351	0.87 (0.77–0.99)	0.034	0.86 (0.75–0.98)	0.022
Co-pathogens present in stool								
*Giardia*								
Absent	Ref							
Present	1.09 (0.94–1.26)	0.249	1.02 (0.87–1.19)	0.839	1.02 (0.91–1.15)	0.633	1.10 (0.97–1.25)	0.134
*Campylobacter*								
Absent	Ref							
Present	1.23 (1.02–1.48)	0.030	1.42 (1.16–1.73)	0.001	1.16 (0.99–1.35)	0.058	1.22 (1.04–1.43)	0.016
*Aeromonas*								
Absent	Ref							
Present	0.78 (0.55–1.10)	0.163	1.26 (0.87–1.81)	0.218	1.08 (0.85–1.38)	0.484	1.26 (0.98–1.63)	0.077
*Shigella*								
Absent	Ref							
Present	0.61 (0.33–1.13)	0.123	0.55 (0.30–1.02)	0.057	0.96 (0.64–1.44)	0.864	1.06 (0.71–1.60)	0.774

Multiple logistic regression was performed for association analysis between dependent and independent variables. The z-score for height-for-age, weight-for-age, and weight-for-height or length were adjusted as continuous variables. We also controlled sites as a cluster effect in the multivariate logistics regression model. aOR = adjusted odds ratio; EPEC = enteropathogenic *Escherichia coli*; ETEC = enterotoxigenic *Escherichia coli*; HAZ = height-for-age z-score; OR = odds ratio; Ref = reference; WASH = water, sanitation, and hygiene; WAZ = weight-for-age z-score; WHZ = weight-for-height z-score.

Multivariate logistic regression analysis of EPEC children revealed less likelihood of EPEC-positive MSD cases in both the 12–23-months and 24–59-months age group (Table 3), whereas in asymptomatic cases, only the 24–59 months age group was less likely to get EPEC infection compared with children aged 0–11 months (Table 4). Asymptomatic EPEC infection is significantly higher in children from lower–middle-class families compared with richest families. Surprisingly, asymptomatic EPEC infection in children was associated with available sanitation facilities and handwashing practices with soap. However, MSD because of EPEC infection had no association with wealth index, sanitation facility, and handwashing practices. EPEC-positive asymptomatic but not MSD children showed a positive association with the presence of *Campylobacter* in stool as a co-pathogen (Tables 3 and 4). The EPEC-positive MSD cases had higher odds of experiencing a loss of skin turgor compared with EPEC-negative MSD cases (Table 3).

### Changes in nutritional status ∼60 days after infection with ETEC or EPEC.

**Figure 5.**
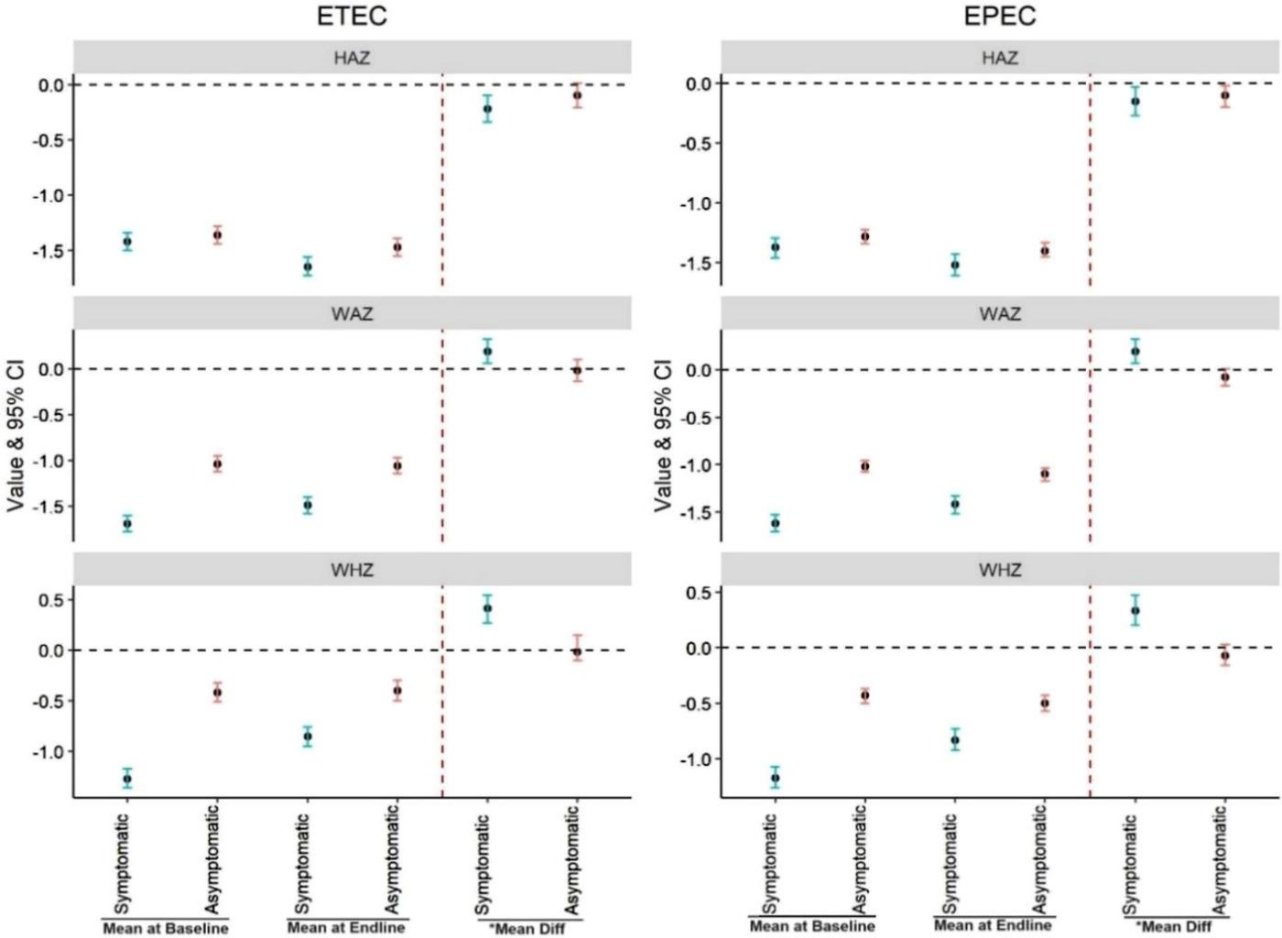
Difference between baseline and end line height-for-age z-score, weight-for-age z-score, and weight-for-height z-score among the children under the age of 5 of enterotoxigenic *Escherichia coli* (ETEC) and enteropathogenic *Escherichia coli* (EPEC) of Global Enteric Multicenter Study (GEMS).

We estimated the mean difference in HAZ, WAZ, and WHZ scores between enrollment and follow-up visits at ∼60 days, stratified by fecal ETEC and EPEC positivity. We demonstrated significant improvements in WAZ scores and WHZ scores over this period in ETEC, as well as EPEC MSD, indicating improved nutritional status. However, significant growth faltering (decreased HAZ score) was noted in both ETEC- and EPEC-positive MSD children (Figure 5). Interestingly, among the asymptomatic ETEC- and EPEC-positive children, we observed the same trend that the HAZ decreased, whereas there were improvements in WAZ scores and WHZ scores from enrollment to follow-up (Figure 5). In adjusted baseline anthropometry, the WAZ score was positively associated with the ETEC pathogen, but the HAZ and WHZ showed a reverse association with ETEC (Table 4). We also examined the relationship between children’s nutritional status, represented by HAZ, WAZ, and WHZ, and the duration of hospital stay over 3 days for cases of MSD associated with ETEC and EPEC pathogens. At baseline, poorer nutritional status was significantly associated with longer hospital stay, particularly for HAZ and WAZ. For instance, children with lower HAZ scores had adjusted ORs (aORs) of 2.56 (*P* = 0.024) for ETEC and 2.27 (*P* = 0.043) for EPEC, whereas WAZ scores also showed significant association with longer stay, with aOR values of 2.87 (*P* = 0.033) for ETEC and 2.44 (*P* = 0.044) for EPEC. However, WHZ scores demonstrated a weaker, nonsignificant relationship at baseline, suggesting that weight-for-height might be a less reliable predictor of hospital duration. By the end line period, these associations were no longer statistically significant for all children who sought care for MSD (Supplemental Table 2).

## DISCUSSION

Secondary analysis from the GEMS large dataset provided us with the unique opportunity to justify the existing epidemiological features of ETEC and EPEC infection as a whole in different age groups. Follow-up visits ∼60 days after enrollment also enabled us to analyze the impact of ETEC and EPEC infection on nutritional status in under-five children.

In this study, we demonstrated that the prevalence of symptomatic ETEC and EPEC infection was just over 11%, whereas asymptomatic ETEC and EPEC infection were 7.4% and 12.1%, respectively. Factors influencing the protection or predisposition of individuals to symptomatic infections have not been explored in the present study. However, in a recent study, it was shown that the composition of the gut microbiome affects the colonization of gastrointestinal pathogens, resulting in a higher bacterial burden in symptomatic individuals with ETEC infection.^18^ The presence of strains with differential expression of ETEC toxins and colonization factors might also explain why some infected children get sick and others don’t.^19^ MSD because of ETEC was found to occur mainly in children 12–23 months of age, whereas symptomatic and asymptomatic children <1 year of age were found to be most susceptible to EPEC infection. In general, ETEC and EPEC frequently caused diarrhea in children younger than 2 years of age.^20^^,^^21^ When further stratified, ETEC was most frequently found in children <1 year in hospitalized patients in India, Bosnia, and Herzegovina, whereas in Brazil, it was detected only in children older than 1 year.^22^^–^^24^ On the other hand, in a study in an urban community in Dhaka, Bangladesh, the proportion of ETEC diarrheas was similar during the first and second years of life.^19^ Similar to our findings, EPEC infection was found to be prevalent in weaned infants under 1 year of age in earlier studies in Brazil and Portugal.^24^^,^^25^ These findings are contrary to a Norwegian study, where infants were more likely to be protected against EPEC infection because of maternal immunity and breastfeeding, and most children started developing diarrhea after 1 year while attending kindergarten.^26^

Exclusive breastfeeding for 6 months was protective against developing diarrhea. Exclusively breastfed children were protected as they were unlikely to take any other foods or fluids likely to be contaminated. This protective effect was less for children on mixed breastfeeding and weaned from their mother’s milk. The protective effect of exclusive breastfeeding has been demonstrated using different study designs, in different settings.^27^^,^^28^

We found that sunken eyes, loss of skin turgor, vomiting, and IV rehydration therapy were the features of ETEC-positive MSD, which are in fact the features of dehydration. One of the GEMS studies, which investigated the burden and etiology of MSD in children aged under five across several countries in sub-Saharan Africa and South Asia, found that children with MSD, including those caused by ETEC, frequently exhibited severe dehydration symptoms and often required IV rehydration.^29^ Additionally, Vidal et al. highlighted the prevalence of ETEC among children with MSD and emphasized the severe dehydration and need for intensive rehydration therapy in these cases.^30^ ETEC-induced diarrheal episodes are generally secretory type and marked by a rapid onset of watery, non-bloody diarrhea, and often accompanied by minimal or no fever. Loss of fluids and electrolytes progressively results in dry mouth, rapid pulse, sunken eyes, and decreased skin turgor, and eventually leads to moderate to severe dehydration, which needs IV hydration.^31^^,^^32^ A very recent study conducted in two geographically distinct rural areas in Bangladesh showed that half of the ETEC diarrheal patients present with severe or some dehydration, vomiting, and abdominal cramp.^33^ Loss of skin turgor and IV rehydration therapy was also found to be associated with EPEC-positive MSD. According to Snehaa et el. (2021), the children affected by EPEC infection presented with vomiting and diarrhea, which led to severe dehydration.^34^

Poor WASH (lack of access to clean water, poor sanitary facilities, and poor personal hygiene practices such as not washing hands with soap before eating, before handling/preparing food, or after defecation) can lead to fecal–oral transmission of infectious entities, in particular, diarrheal pathogens.^27^^,^^28^^,^^35^^–^^38^ In line with the general notion, less frequency of use of tube well as drinking water source, handwashing after defecation and before cooking in ETEC- and/or EPEC-positive MSD, and/or asymptomatic children compared with uninfected children was self-explanatory. On the contrary, use of treated water was more in ETEC- negative group than positive group and there are a number of reasons why treated water might not always prevent diarrheal episodes. Effectiveness of water treatment methods is dependent on how sincerely people comply with the treatment. Water that was initially safe can become contaminated with pathogens during transport, storage, or handling at usage point, and organisms may be resistant to the method of treatment. Also, improved sanitation facilities, soap for handwashing, handwashing before nursing a child, preparing a baby’s food, or after cleaning a child who defecated were found more in infected than uninfected groups, which came to us as a surprise. It is worth mentioning that in regression analysis, the use of tube well or treated water, and handwashing after defecation, after cleaning a child who defecated before cooking, and before nursing a child or preparing baby’s food had no association with MSD or asymptomatic infection. Only available sanitation facilities and handwashing practices with soap had an association with asymptomatic EPEC infection. WASH practices are potentially influenced by the socioeconomic condition and education status of the households.^39^ When the wealth index of our study population was considered, infection with ETEC/EPEC in asymptomatic children was found to be more common in children from lower-middle to middle-class households compared with the richest families. Among the included children, whose mothers had no formal education were more likely to experience diarrheal episodes compared to children whose mother received formal education, which supports the findings of a cross-sectional study where they have mentioned that primary education of caregiver as mother was significantly associated with lower risk of diarrhea in children <5 years.^40^ Also, we have found that symptomatic ETEC and EPEC, and asymptomatic ETEC infections in children were more likely to be associated with illiterate mothers. On the other hand, asymptomatic EPEC infection was less likely to be related to mother’s illiteracy.

Previous studies supported that, after causing acute diarrhea followed by clinical recovery, some enteric pathogens like *Campylobacter* are excreted asymptomatically for many days or even weeks. It is also observed that children without diarrhea may excrete pathogens from previous episodes of diarrhea.^41^ The presence of *Campylobacter* in stool samples as a co-pathogen was frequently detected in both asymptomatic and MSD children, highlighting the possibility of complex interactions between these pathogens.^42^^,^^43^ The co-occurrence of *Campylobacter* with EPEC and ETEC validates our study finding that asymptomatic ETEC- and EPEC-positive children showed a positive association with *Campylobacter* in stool as a co-pathogen.^44^ According to Kotloff et al., rotavirus, *Cryptosporidium*, *Shigella,* and ETEC are mostly responsible for diarrheal illness in developing countries.^20^ More than one pathogen was detected in 65% of the fecal sample with acute gastroenteritis in Rwanda and Zanzibar and a positive association was found between virulence factor genes in EPEC and *Shigella,* whereas we found a negative association between asymptomatic EPEC infection with *Shigella.*^45^ After adjusting for other co-pathogens, *Campylobacter* was a common co-pathogen in the case of both MSD- and asymptomatic ETEC-positive children. It coincides with the result of another study showing the other top five pathogens identified with *Campylobacter* infections were ETEC, *Aeromonas*, EAEC, cryptosporidium, and *Giardia lamblia,* and that *Campylobacter* infections, whether presenting with symptoms or not, have been linked to stunted growth in children under the age of 5 in South Asia.^42^ Another GEMS study found *Aeromonas* as a significant contributor to MSD, particularly in Pakistan and Bangladesh, and its frequent co-occurrence with *Shigella,* which highlights our findings that these pathogens can also be present in ETEC-negative children.^46^

The noteworthy observation of our study was that children with ETEC diarrhea demonstrated significant improvements in WAZ scores and WHZ scores. Additionally, significant improvement in WHZ scores in EPEC-infected children was also revealed. Children with diarrhea might have acute weight loss because of anorexia, reduced food intake, and dehydration, which, after discharge, may have resumed with normal dietary intake or receiving nutritional supplement (zinc), which could have improved their WAZ and WHZ scores.^47^ However, significant growth faltering (HAZ score) was noted in children with ETEC and EPEC MSD at the time of ∼60 days follow-up. Asymptomatic ETEC- and EPEC-infected children also experienced growth faltering at follow-up visits, which coincides with the findings of Das et el. that pathogenic variants of *E. coli* had a negative association with child growth.^8^ Another study conducted among Bangladeshi malnourished children also reported that children with EPEC had a significant negative association with linear growth and underweight.^9^ A study conducted in 79 LMICs, showed that ETEC and *Shigella* are the two significant pathogens that contribute to a higher risk of stunting and mortality because of MSD, and stunting resulted in a 24% rise in mortality rates.^48^ According to Black et al., diarrhea associated with ETEC had a significant negative effect on bimonthly weight gain of children in rural Bangladesh.^21^ A meta-analysis of 34 sub-Saharan African countries found that ETEC diarrhea was associated with 1.5 times higher odds of stunting (low height-for-age) and 1.4 times higher odds of underweight (low weight-for-age) among children under 5 years old.^49^ Such scenarios have been observed more often in malnourished children who experienced a fatal outcome because of prolonged or persistent diarrheal episodes. We observed a significant association between length of hospital stay and abnormal nutritional status. The length of hospital stays progressively increased with deterioration of nutritional status. Such associations are indicative that any increase in length of hospital stay may progressively cause deterioration of nutritional status, particularly stunting and underweight. The attending physician is in a key position to assess nutritional status of hospitalized patients. If malnutrition can be documented on hospital admission, attempts can be made to reverse the malnutrition and hopefully diminish the length of hospital stay. This may also have policymaking implications such as providing calorie-rich diet to the hospitalized children with acute watery diarrhea to hinder their declining trend in nutritional status.

It is also observed that children with atypical EPEC are significantly associated with prolonged diarrhea.^26^ On the contrary, children with MSD were three times more likely to be acutely malnourished than the asymptomatic control children and the odds of having MSD were seven times higher in these children compared with children with better nutritional status.^50^ Severe diarrhea did not seem to affect the nutritional status of the children at follow-up visits, although children with wasting at enrollment were nine times more likely to have diarrhea than those without wasting. Preventive and therapeutic strategies for improving the nutritional status of children and the algorithm for diarrhea management in children should be reinforced.

The strengths of the study were the unbiased sampling method, use of randomization procedure in the selection of asymptomatic children, large sample size, and high-quality laboratory performance. Data collection by administering field-tested data collection tools, as well as measurements of nutritional status by a follow-up visit of children at the household level, significantly contributed in assessing and explaining growth outcomes in study children.

Limitations of this study include a single follow-up visit after 60 days of enrollment, which may not show the actual effect on growth. Baseline weight was taken after rehydration, but for a few children who left the facility before hydration, their baseline weight was taken before hydration, which might interfere with baseline and end line growth status. Our analysis included information on co-infection that might interact and influence the child nutritional status. Also, the study lacked related information that could confound the results of the study, such as data on maternal body mass index, gestational age, birthweight, vitamin and micronutrient status, and inflammatory biomarkers, which are the hallmarks of enteropathy of the children, as well as their prevailing immunocompromised status like HIV. Also, we didn’t know which pathotypes of *E. coli* were predominant in cases and controls positive for both ETEC and EPEC, as colonies were pooled and the pathotypes of *E. coli* were detected using PCR.

## CONCLUSION

As no simple diagnostic tests are currently available for detection of ETEC and EPEC, the basis of clinico-epidemiological characteristics would be helpful for the early diagnosis and management of ETEC and EPEC diarrhea. Children with ETEC-MSD had more sunken eyes, while EPEC-MSD children presented with loss of skin turgor requiring IV rehydration. Significant growth faltering among MSD children infected with ETEC and EPEC during follow-up highlights the urgent need for preventive measures, including vaccine trials targeting ETEC and EPEC diarrhea in children >5 years of age.

## Supplemental Materials

10.4269/ajtmh.24-0347 Supplemental Materials

## Data Availability

A publicly available GEMS dataset was analyzed in this study. These data can be obtained here: Clinical Epidemiology Database Resources (ClinEpiDB) (https://clinepidb.org/ce/app/record/dataset/DS_841a9f5259). After the thorough review and approval process by the ClinEpiDB study team, we have obtained official data access from ClinEpiDB, the responsible entity for managing the GEMS data repository.
