# Supplementary material for: Clinico-Epidemiological Characteristics of Symptomatic and Asymptomatic Enterotoxigenic and Enteropathogenic Escherichia Coli Diarrhea and Impact on Child Growth
Source: Am J Trop Med Hyg. 2025 May 13;113(1):120–33. doi: 10.4269/ajtmh.24-0347 (PMC12225550; doi:10.4269/ajtmh.24-0347)
Supplement: Supplemental Materials [file tpmd240347.SD1.pdf]

S1 Table: Association between Growth and Duration of Hospital Stay of MSD

Anthropometric	Study Period	ETEC		EPEC	
		Coefficient	*p*-value	Coefficient	*p*-value
HAZ	Baseline	-0.10 (-0.16, -0.05)	<0.001	-0.09 (-0.14, -0.04)	0.001
	Endline	-0.11 (-0.17, -0.05)	<0.001	-0.08 (-0.18, -0.01)	0.075
WAZ	Baseline	-0.13 (-0.18, -0.07)	<0.001	-0.14 (-0.20, -0.07)	<0.001
	Endline	-0.10 (-0.18, -0.03)	0.005	-0.08 (-0.20, 0.03)	0.159
WHZ	Baseline	-0.09 (-0.16, -0.04)	0.001	-0.13 (-0.20, -0.06)	<0.001
	Endline	-0.07 (-0.15, 0.02)	0.112	-0.06 (-0.17, 0.05)	0.325

The table examines the relationship between child growth indicators (HAZ, WAZ, and WHZ) and the duration of hospital stay for children with moderate-to-severe diarrhea (MSD), focusing on two pathogens, ETEC and EPEC, across baseline and endline periods. Overall, lower Z-scores, which reflect poorer nutritional status, are significantly associated with longer hospital stays, particularly at baseline. For both pathogens, HAZ and WAZ consistently show significant negative associations at baseline, indicating that children with lower height-for-age and weight-for-age scores tend to have longer hospital stays. These associations are weaker or non-significant at endline, particularly for WHZ, which shows the least significant relationship, suggesting that weight-for-height may be less predictive of hospital duration over time.

S2 Table: Association between ETEC diarrhea and EPEC diarrhea with non-breastfeeding child by age group

	ETEC		EPEC	
Age (Months)	OR	*p*-value	OR	*p*-value
0-11	1.24	0.169	1.17	0.239
12-23	1.08	0.334	0.95	0.576
24-59	1.51	0.001	0.94	0.593

Separate analyses were conducted for each age group. Breastfeeding is a categorical variable (breastfed, non- breastfeed) where breastfed is the reference group

Table S2 shows the relationship between non-breastfeeding and the likelihood of developing diarrhea caused by Enterotoxigenic Escherichia coli (ETEC) and Enteropathogenic Escherichia coli (EPEC) across different age groups. In children aged 24-59 months, non-breastfeeding is significantly associated with a higher risk of ETEC-related diarrhea (OR 1.51, p=0.001), suggesting that non-breastfeeding may increase susceptibility to ETEC infections in this age group. No significant association is found for EPEC diarrhea across any age group, indicating that breastfeeding status may have a stronger influence on ETEC infections than on EPEC infections, particularly in older children.

S3 Table: Number of participants with ETEC or EPEC positive in MSD and Asymptomatic group

**Total**	**MSD (Symptomatic)**	**Control (Asymptomatic)**
ETEC (+) and EPEC (-) =1873 (9.39 %)	ETEC (+) and EPEC (-) =991 (11.81 %)	ETEC (+) and EPEC (-) =882 (7.64 %)
ETEC (-) and EPEC (+) = 2461 (93.57 %)	ETEC (-) and EPEC (+) = 972 (92.75 %)	ETEC (-) and EPEC (+) = 1489 (94.12 %)

Table S3 shows number of participants either positive for ETEC or EPEC in stool culture. Among all participants, 1,873 (9.39%) were positive for ETEC and negative for EPEC. Within the MSD group, 991 (11.81%) tested positive for ETEC, whereas 882 (7.64%) exhibited ETEC positivity in the asymptomatic group. A total of 2,461 (93.57%) participants were negative for ETEC and positive for EPEC. Among the symptomatic MSD group, 972 (92.75%) were EPEC positive, compared to 1,489 (94.12%) in the asymptomatic group.
